# Supplementary material for: Directed self-assembly of liquid crystalline blue-phases into ideal single-crystals
Source: Nat Commun. 2017 Jun 16;8:15854. doi: 10.1038/ncomms15854 (PMC5481765; doi:10.1038/ncomms15854)
Supplement: Supplementary Information [file ncomms15854-s1.pdf]

Type of file: PDF
Size of file: 0 KB
Title of file for HTML: Supplementary Information
Description: Supplementary Figures

Type of file: PDF
Size of file: 0 KB
Title of file for HTML: Peer Review File
Description:

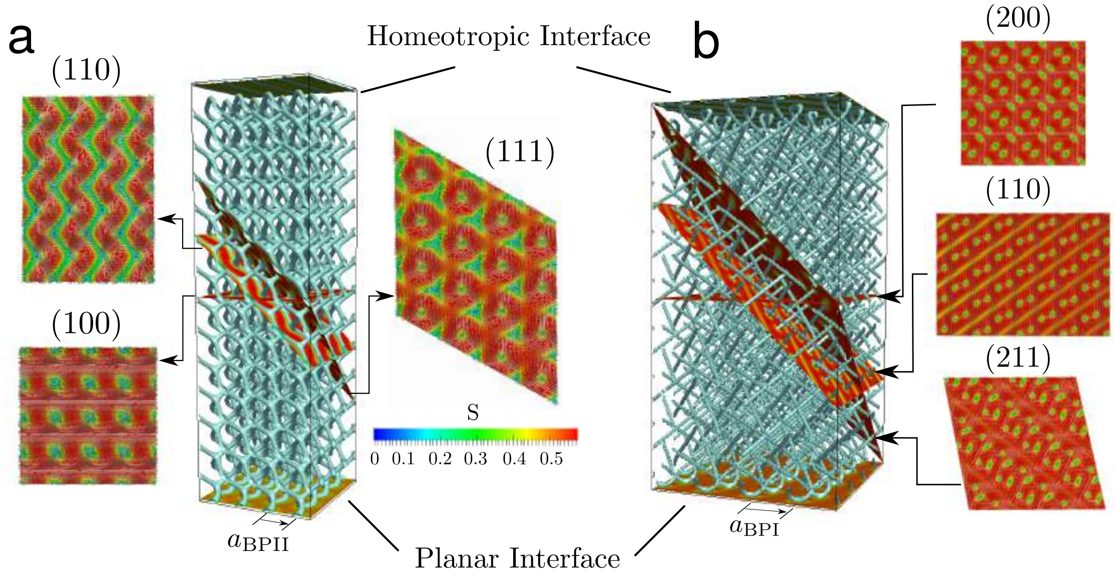

Supplementary Figure 1. Topological defects and crystallographic planes of BPs. Defect structure of (a) $BPII_{(100)}$ and (b) $BPI_{(200)}$. Line topological defects are visualized as blue isosurfaces of the nematic order parameter. The corresponding crystallographic planes, for which light reflection is allowed, are also shown. For each crystallographic plane the color depends on the local value of the scalar order parameter.

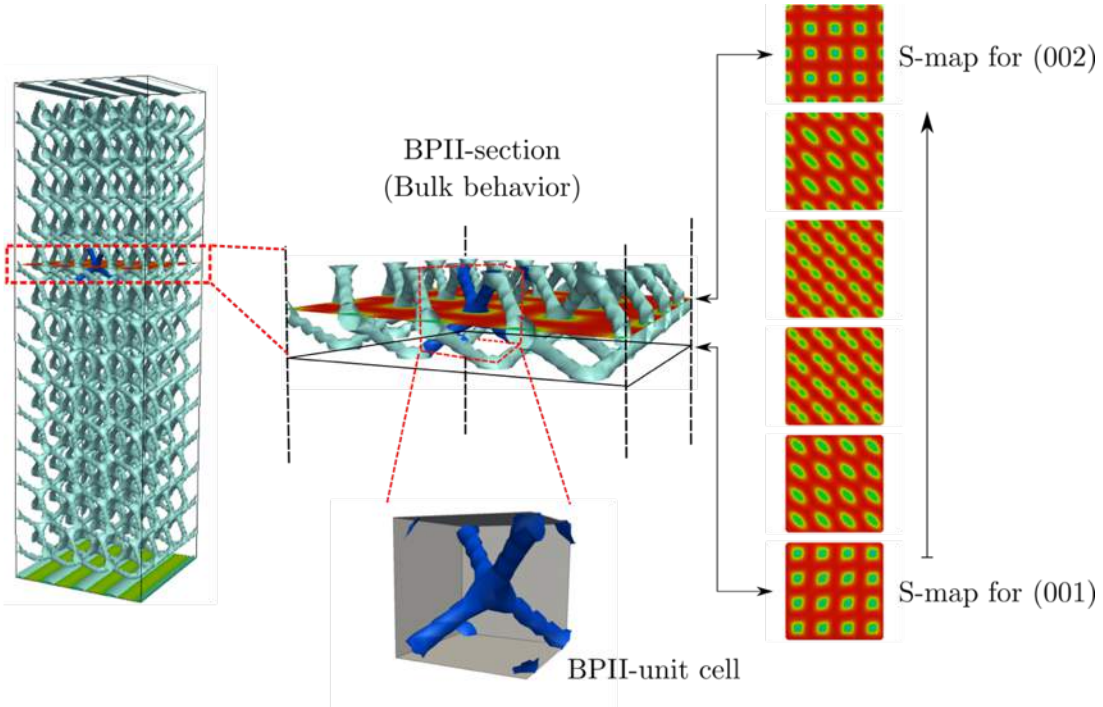

Supplementary Figure 2. S-map behavior of a $BPII_{(100)}$ in bulk. S-map for different crystallographic planes in bulk, these planes are parallel to the (001). As can be seen from the figure, the S-map is useful to indicate regions where the BP-disclination lines are crossing these planes but do not give information about the preferred molecular alignment.

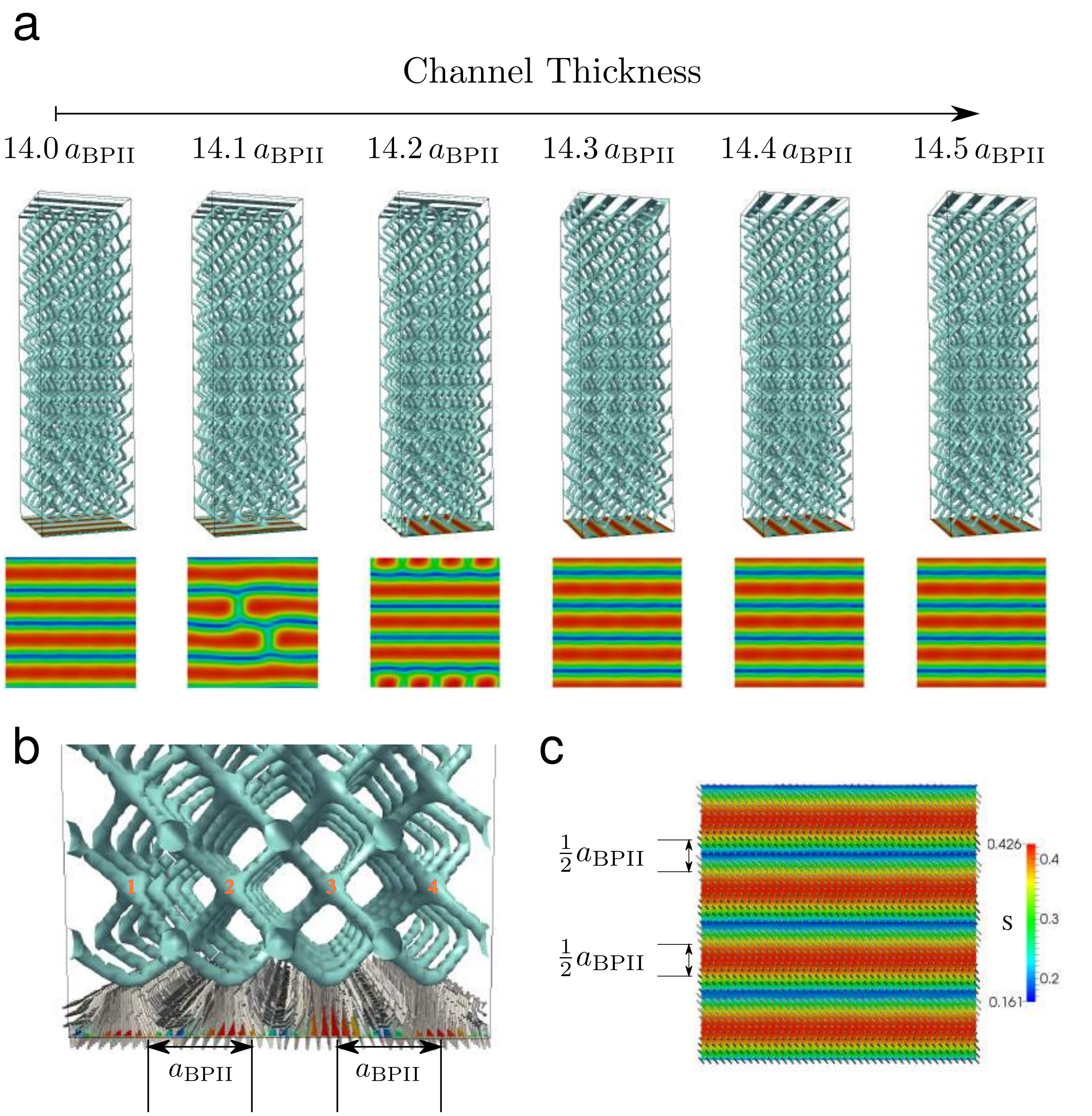

Supplementary Figure 3. S-maps of a BPII$_{(100)}$ at the homeotropic interface in channels with different thickness. (a) BPII$_{(100)}$ disclination lines and S-maps for different channel thickness. (b) BPII$_{(100)}$ disclination lines at the proximity of the surface and (c) director field at the homeotropic interface. There are two significant differences with respect to the situation in bulk. First, as shown in (a), the symmetry of the S-map remains basically unchanged as the channel thickness increases. Second, because of the homeotropic

anchoring conditions, the S-map at the interface can be associated to the preferred molecular alignment at the immediate proximity above it, as can be observed from (b); i.e., the order parameter is high if the molecular alignment of the BP above the interface is perpendicular, and is low when the molecular alignment shows a planar tendency, as shown in (c).

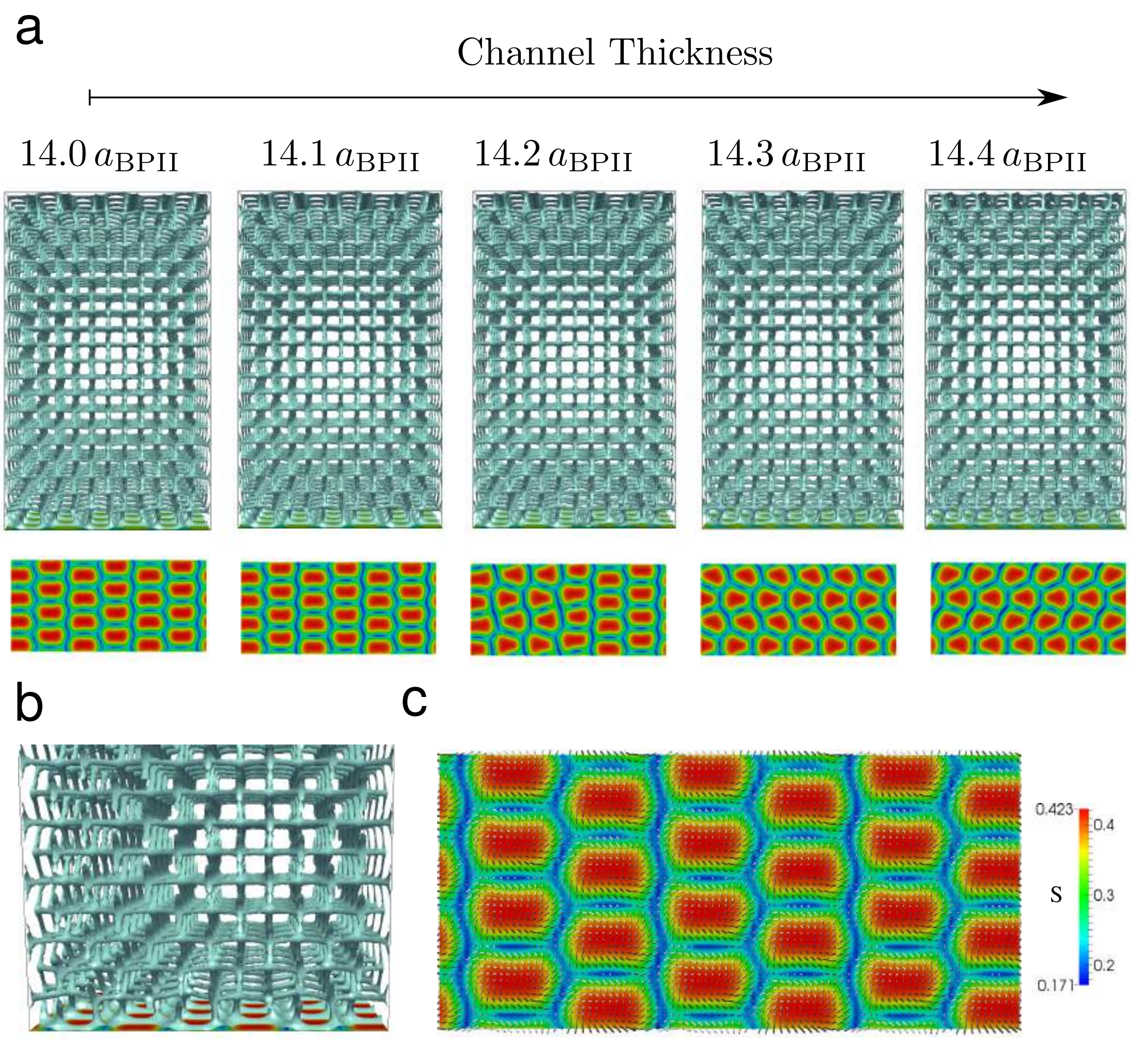

Supplementary Figure 4. S-maps of a $\mathrm{BPII}_{(110)}$ at the homeotropic interface in channels with different thickness. (a) $\mathrm{BPII}_{(110)}$ disclination lines and S-maps for different channel

thickness. Although there is a change in the shape of the red regions, there remain their hexagonal distribution. Additionally, the main role of the patterns that are proposed is to seed the preferred BP-crystallographic orientation. (b) $BPII_{(110)}$ disclination lines at the proximity of the surface and (c) director field at the homeotropic interface.

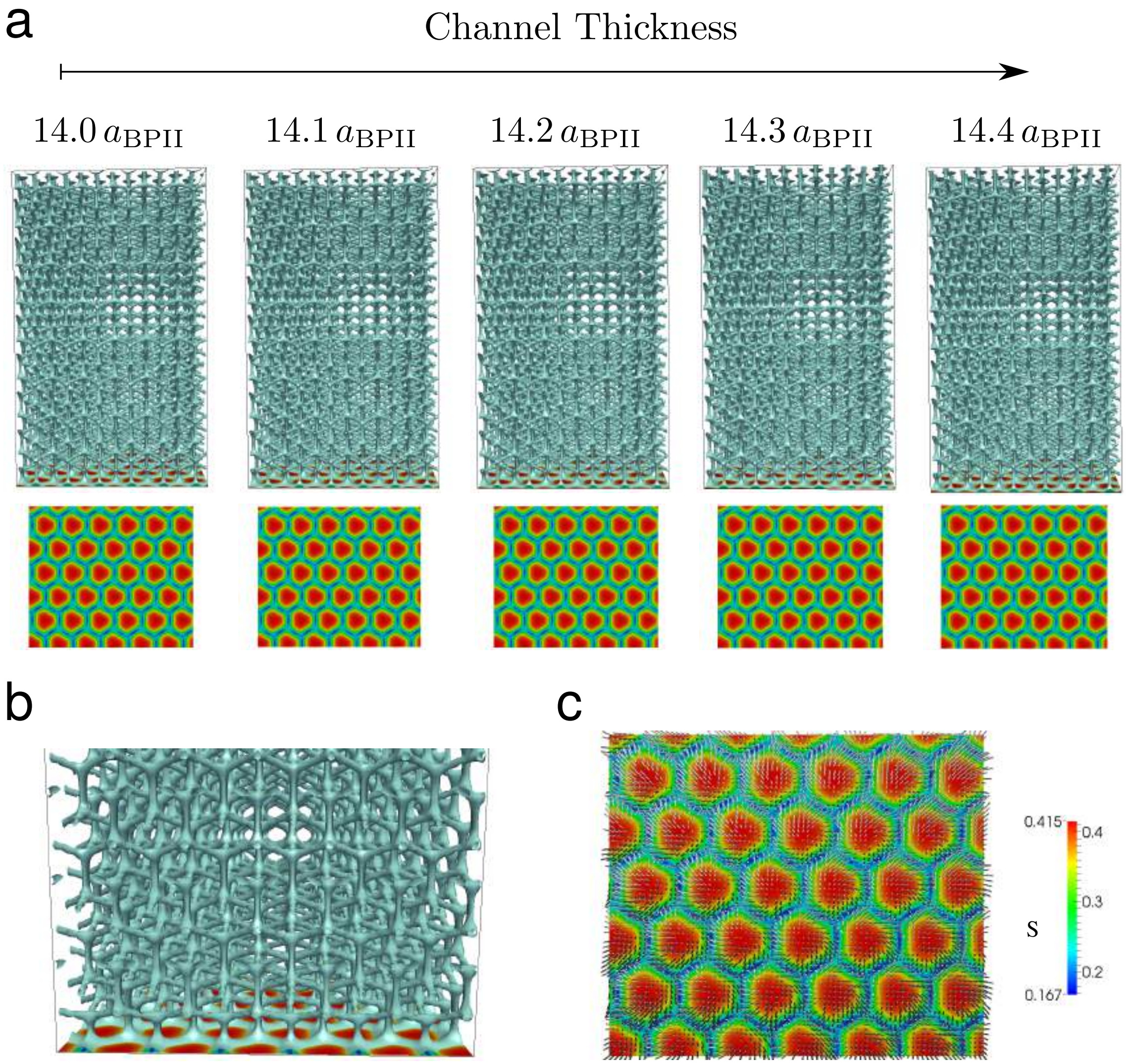

Supplementary Figure 5. S-maps of a $BPII_{(111)}$ at the homeotropic interface in channels with different thickness. (a) $BPII_{(111)}$ disclination lines and S-maps for different channel thickness. For these case, we do not observe any change in the pattern shape and

distribution as the channel thickness varies. (b) $BPII_{(111)}$ disclination lines at the proximity of the surface and (c) director field at the homeotropic interface.

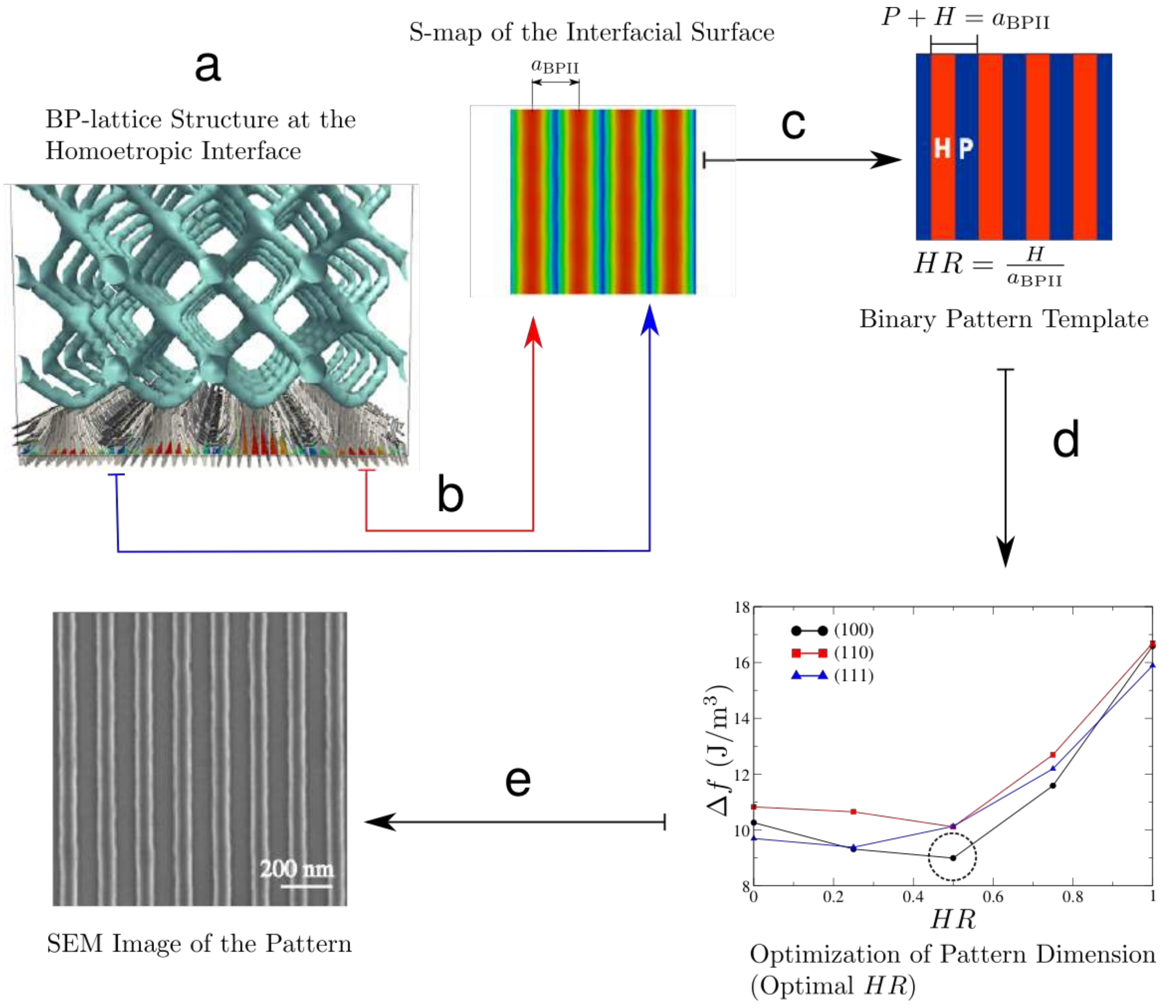

Supplementary Figure 6. Strategy to produce a single crystal of $BPII_{(100)}$. (a) Continuum simulations of a $BP_{(100)}$ under uniform homeotropic conditions are performed. (b) The S-map at the interface is correlated with the preferred molecular alignment. (c) The S-map is mapped into a binary pattern made of planar and homeotropic regions. (d) Theoretic field calculations are preformed to determine the optimal dimensions of the homeotropic

and planar regions. (e) Once the pattern is optimized, the obtained information is used to prepare it by chemical treatments.

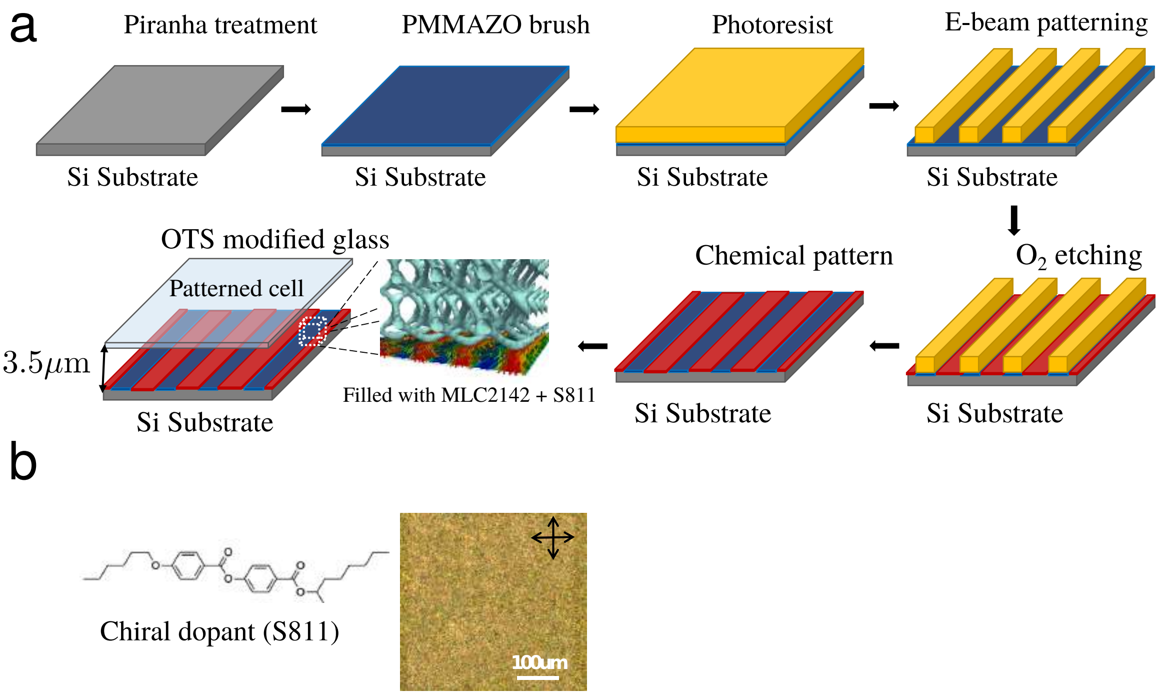

Supplementary Figure 7. Fabrication of chemically patterned surfaces. **(a)** Schematic representation of the process used for producing chemically patterned surfaces. **(b)** The chiral dopant S811 is added to the liquid crystal MLC2142 to produce the chiral liquid mixture that is used in our experiments. At room temperature we observe a cholesteric phase texture, as shown in the micrograph on the right.

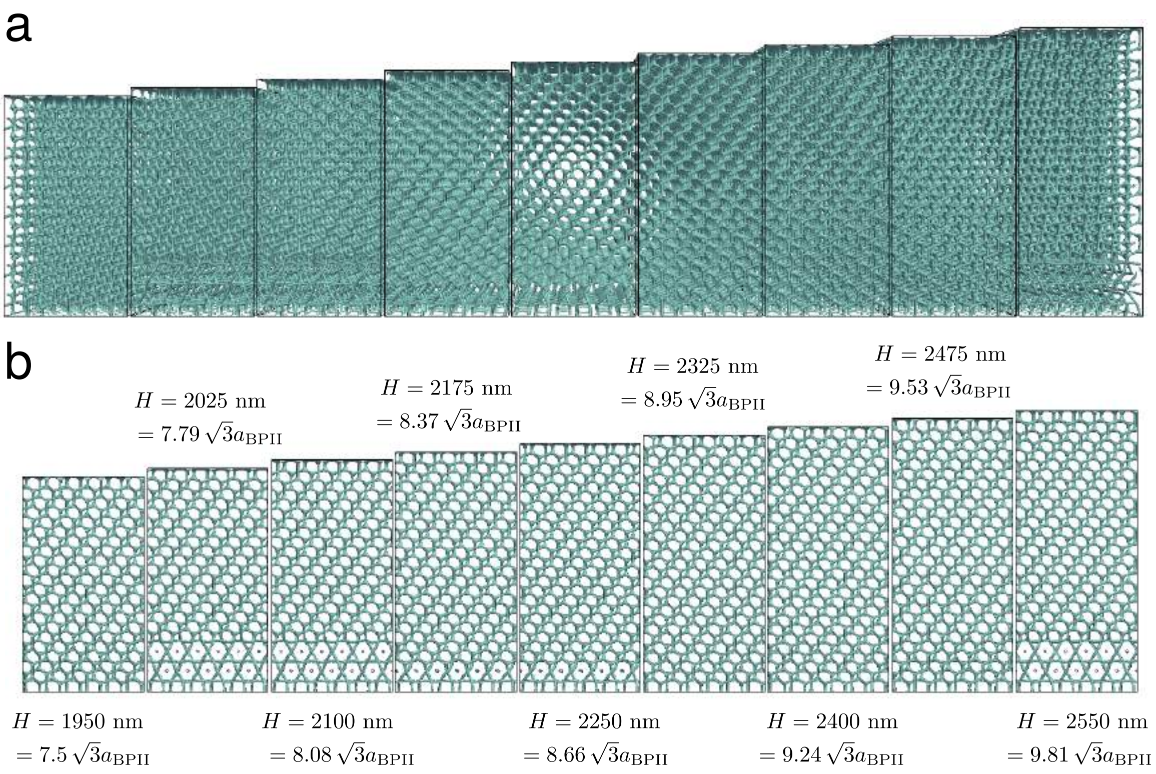

Supplementary Figure 8. Local hexagonal blue-phase induced by confinement (a) Line defect structure of a $BPII_{(111)}$ for different channel thicknesses and (b) the corresponding 2D projection. In some cases, the structure adopts a structure that is consistent with a hexagonal blue-phase symmetry. The channel's thickness is given in nanometers and in terms of the distance of two subsequent (111) planes, i.e. $\sqrt{3}\ a_{BPII}$.

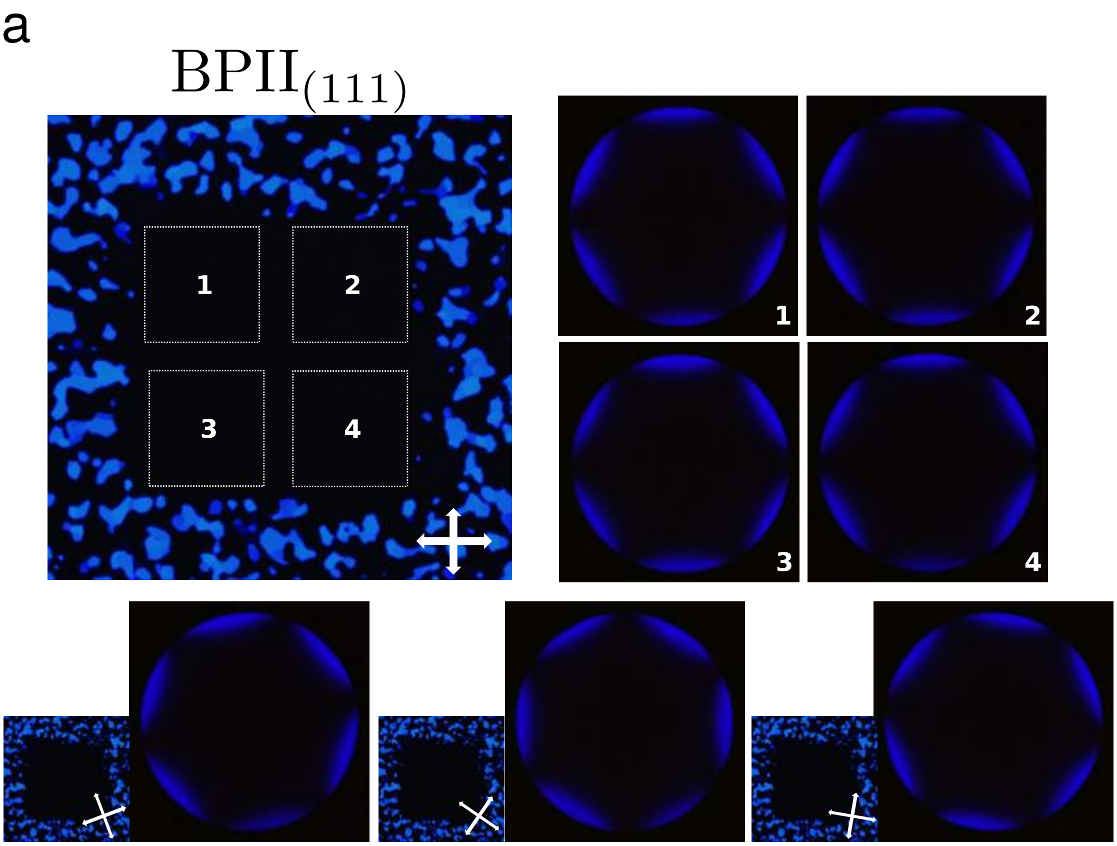
a
BPII$_{(111)}$
1
2
3
4
1
2
3
4

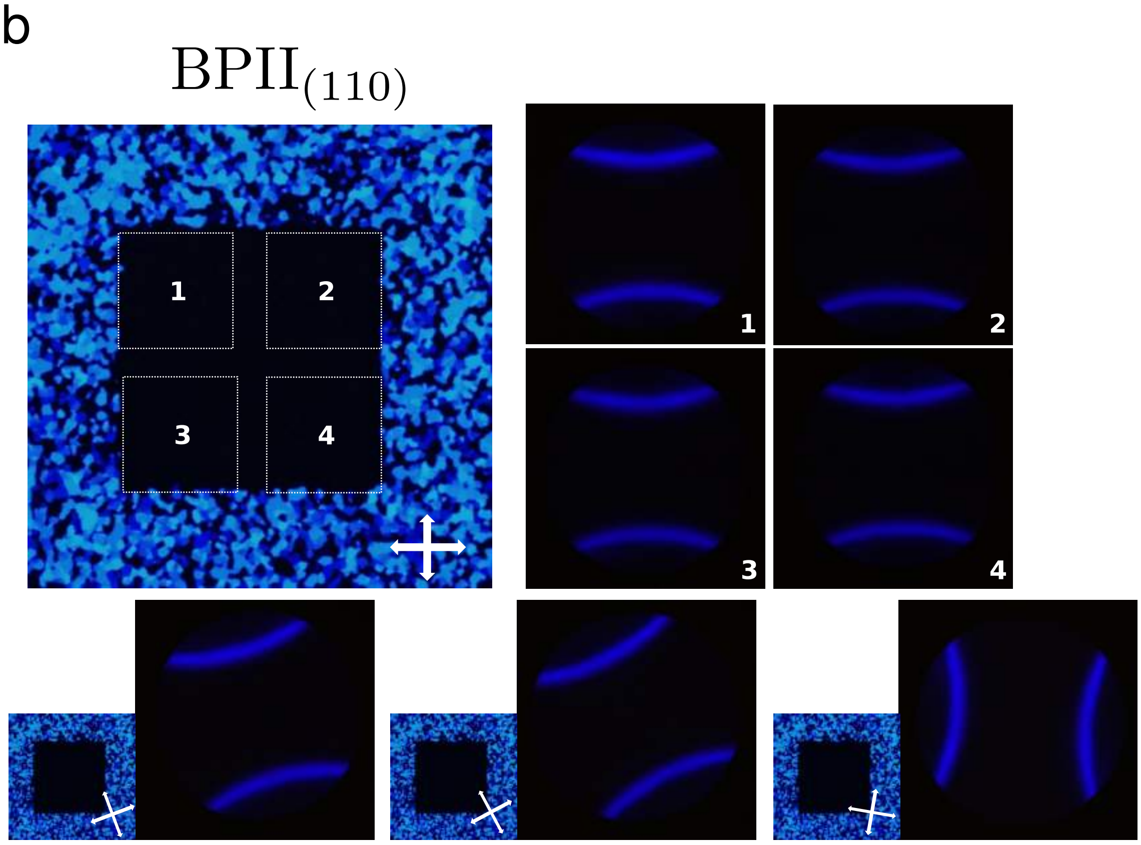
b
BPII$_{(110)}$
1
2
3
4
1
2
3
4

Supplementary Figure 9. Kossel diagrams in a single-crystal and a polyscrystalline BPII. Cross-polarizer micrographs of (a) $BPII_{(111)}$ and (b) $BPII_{(110)}$ taken in reflection mode, they appear dark because the wavelength of the reflected light is out of the visible spectrum. Kossel diagrams are obtained to confirm the lattice orientation. Additionally, these diagrams are taken while "scanning" different positions of the patterned area; we found that they remain identical which is an indication of the uniform order of the BP sample. In polycrystalline samples, Kossel diagrams rotates while scanning different positions, as shown below each panel.

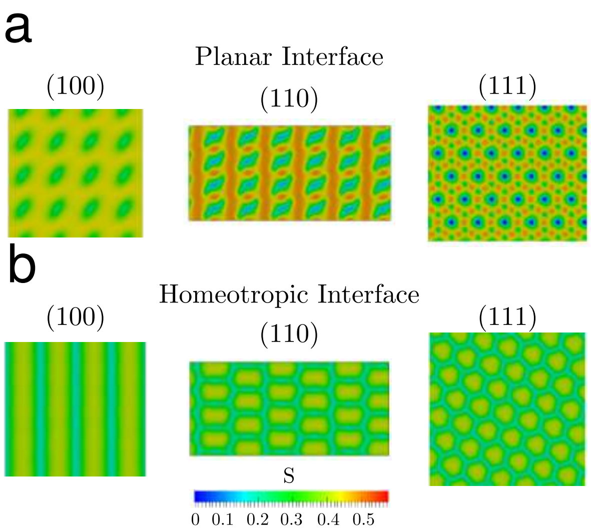

Supplementary Figure 10. S-maps from homeotropic and planar interfaces. S-maps for different crystallographic orientations at the (a) planar and (b) homeotropic interfaces. In this work, patterns are based on the S-map behavior at homeotropic interfaces since they show the easiest symmetries to engineer.

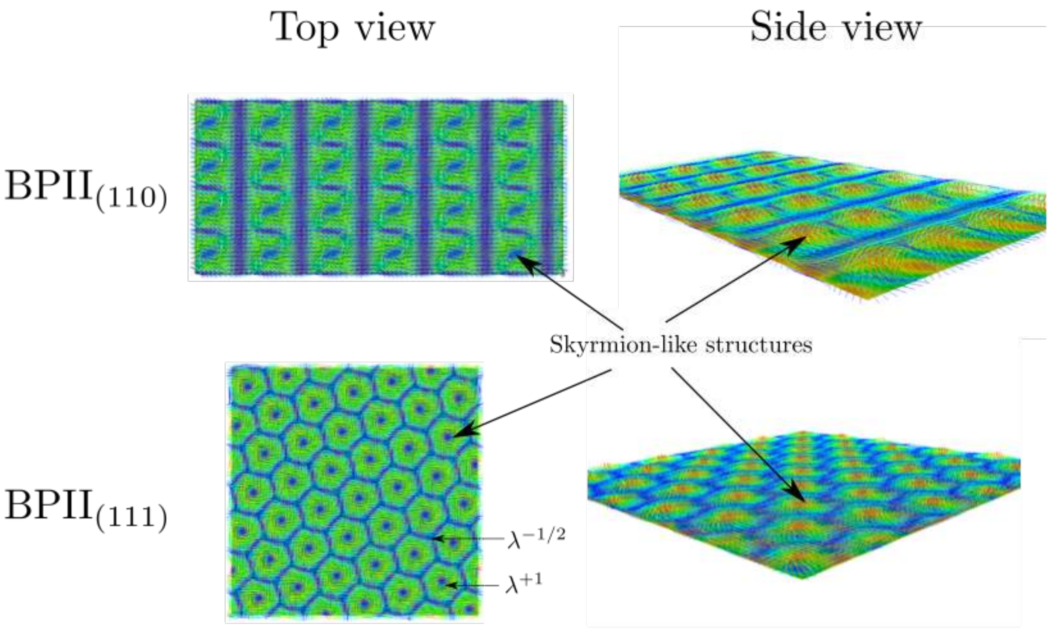

Supplementary Figure 11. Skyrmion-like structures formed by $BPII_{(110)}$ and $BPII_{(111)}$ molecules in the proximity of uniform planar interfaces. The global molecular behavior for each case is characterized by an ordered distribution of positive and negative charges of topological defects.
